# A population-based twin study of the symptomatic diagnostic criteria for major depression that occur within versus outside of major depressive episodes

**DOI:** 10.1017/S0033291723001241

**Published:** 2023-11

**Authors:** Kenneth S. Kendler, Steven H. Aggen

**Affiliations:** 1 Virginia Institute for Psychiatric and Behavioral Genetics, Richmond, VA, USA; 2 Department of Psychiatry, Virginia Commonwealth University School of Medicine, Richmond, VA, USA

**Keywords:** Depressive symptoms, diagnosis, major depression, within and outside of episodes

## Abstract

**Background:**

Are genetic risk factors for current depressive symptoms good proxies for genetic risk factors for syndromal major depression (MD)?

**Methods:**

In over 9000 twins from the population-based Virginia Adult Twin Study of Psychiatric and Substance Use Disorders, the occurrence of all nine DSM symptomatic criteria for MD in the last year was assessed at personal interview and then grouped by their temporal co-occurrence. The DSM criteria which occurred outside (OUT) *v.* inside of (IN) MD episodes were then separated. We calculated tetrachoric correlations for OUT and IN depressive criteria in monozygotic (MZ) and dizygotic (DZ) pairs and fitted univariate and bivariate ACE twin models using OpenMx.

**Results:**

The mean twin correlations (±95% CIs) for IN depressive criteria were substantially higher than for OUT depressive criteria in both MZ [+0.35 (0.32–0.38) *v.* 0.20 (0.17–0.24)] and DZ pairs [0.20 (0.17–0.24) *v.* 0.10 (0.04–0.16]. The mean IN–OUT cross-correlation in MZ and DZ pairs was modest [+0.15 (0.07–0.24) and +0.07 (0.03–0.12)]. The mean heritability estimates for the nine In *v.* Out depressive criteria was 0.31 (0.22–0.41) and 0.15 (0.08–0.21), in MZ and DZ pairs, respectively. The mean genetic correlation between the nine IN and OUT depressive criteria was +0.07 (−0.07 to 0.21).

**Conclusions:**

Depressive criteria occurring outside depressive episodes are less heritable than those occurring within. These two ways criteria can manifest are not closely genetically related. Current depressive symptoms – most of which are occurring outside of depressive episodes – are not, for genetic studies, good proxies for MD.

In the field of mental health, the term *depression* has many meanings of which two are predominant. The first is a syndromal diagnosis of an episode of major depression (MD) meeting diagnostic criteria, for example, those defined in DSM-5 (American Psychiatric Association, 2013). In research settings, such episodes are most commonly assessed by a clinically trained interviewer applying one of several structured or semi-structure psychiatric interviews. The goal of such data collection is typically to determine if individuals meet diagnostic criteria for MD over a particular time period, most frequently the last year or their lifetime.

The second common form of ‘*depression*’ is current depressive symptoms typically reported over the last few weeks assessed by one of numerous self-report questionnaires (Fried, 2016) which include the PHQ9 (Kroenke, Spitzer, & Williams, 2001) which lists the symptomatic DSM-5 criteria for MD. Sometimes such scales are used in treatment studies to assess symptomatic change, but our focus here will be on their use in general population samples (e.g. college students, community cohorts) with the goal of determining the level of current depressive symptomatology. Using traditional methods, collecting information on syndromal MD is much more resource intensive than assessing self-report of current depressive symptoms.

Driven by the increasing availability of molecular genetic data on large samples of subjects, considerable debate has arisen about the relationship between these two constructs of depression. Two broad positions have emerged. The first or *convergent* position suggests that these two constructs are, from an etiologic perspective, closely inter-related, with syndromal MD representing a more severe form of the same dimension of liability that is assessed by self-report depressive symptom questionnaires. Advocates for this position typically suggest that, given the greater convenience and low cost, current depressive symptoms can function as a valid proxy for lifetime MD measures in a range of research applications including genetic studies.

The second or *divergent* position argues that these two constructs for depression are qualitatively different. One assesses depressive symptoms that co-occur in temporally defined depressive episodes which reflect the clinical course of MD while the other assesses many of the same symptoms that occur currently and typically not as a part of depressive episode. While advocates for this position typically accept that the two constructs are weakly inter-correlated, they argue that depressive symptoms are not, for research purposes, an adequate proxy for lifetime MD.

Two arguments for the divergent position are worth explicating. The first concerns the key difference in the context of symptom occurrence. In the general population, there are many reasons for individuals to have sleep or eating difficulties, changes in weight, tiredness, or problems with concentration that have little or nothing to do with being depressed. People often gain weight at the holidays, become tired when working too hard at a job or caring for young children, and sleep poorly because of job or school-related anxiety or because of pain from various sources. While such extraneous causes for depressive symptoms could occur for depressive criteria assessed for lifetime MD, they are likely to be less influential because of the presence of an active pathophysiological and/or mental process causing the depressive syndrome as reflected in the many other co-occurring symptoms and signs. Second, meeting the full DSM diagnostic criteria requires the presence of a substantial number of co-occurring depressive symptoms lasting for a sustained period of time. By contrast, the distributions of most depressive symptom scores are substantially rightward (positively) skewed with the mean and median considerably below the cut-off used to indicate clinical depression (Kendler, Heath, Martin, & Eaves, 1986; Knight, Waal-Manning, & Spears, 1983; Silberg et al., 1990; Teri, 1982). Thus, most population variance in these scales occurs at the low end of depressive symptom counts which might reflect qualitatively different etiological factors than those captured by the more severe symptoms endorsed in syndromal MD.

In this report, we evaluate empirically these two positions on the relationship between MD and depressive symptoms. We examine a large population-based twin sample where all the symptomatic criteria for MD in the last year were assessed at personal interview and then grouped by their temporal co-occurrence into those which occurred outside (OUT) *v.* inside of (IN) MD episodes. The convergent hypothesis would predict similar heritabilities for the two forms of depressive symptoms and strong genetic inter-correlation. By contrast, the divergent hypothesis would predict higher heritabilities for depressive symptoms within MD episodes and a relatively low genetic correlation between the symptoms that occurred within *v.* outside depressive episodes.

## Methods

We here attempt to address the role of familial/genetic factors in symptomatic criteria for MD that do *v.* do not occur in the context of an MD episode. In the Virginia Adult Twin Study of Psychiatric and Substance Use Disorders (VATSPSUD) (Kendler & Prescott, 2006), over 9000 twins were personally interviewed by trained mental health professions including the assessment of all nine DSM-IV A criteria depressive symptoms (American Psychiatric Association, 1994) over the year prior to the date of the interview. At the conclusion of that interview section, if more than one symptom was reported, the respondent was asked to group the symptoms that temporally co-occurred. That permitted us to divide the occurrence of these symptomatic criteria into two groups – those we call ‘IN’ which occurred as part of a syndromal MD episode meeting the DSM criteria for positive case status and those we call ‘OUT’ which occurred either on their own or with a few other symptoms that fell below meeting criteria for a depressive episode.

We then assessed the similarity of the IN and OUT depressive criteria by examining their tetrachoric correlation in MZ and DZ twins, their cross-correlation in twins, and then, by univariate and bivariate model-fitting, their heritability and genetic correlation. We suggest that advocates of the Convergent position on depression would predict that the twin correlations and heritability would be similar in magnitude for the IN and for the OUT depressive criteria and the IN–OUT correlation across twins would be high because they result from the same familial/genetic factors.

By contrast, advocates for the divergent position would suggest that twin correlations and heritability would be lower for the OUT than for the IN depressive criteria. That is because random environmental events not shared with the cotwin are likely to have a greater impact on OUT than IN depressive symptoms. Furthermore, these advocates would predict relatively low IN–OUT cross-correlations in twin pairs because they would suggest IN and OUT criteria do not, by and large, reflect the same familial/genetic liabilities and low genetic correlations.

### Sample

Sample data for this study came from detailed interviews administered to two related cohorts from the VATSPSUD. Briefly, the Virginia Twin Registry was established in 1980 using a population-based database of multiple births records occurring in the Commonwealth of Virginia held by the Virginia Department of Health Statistics since 1918. Contact details for twins were obtained by matching names and birth dates to state records, such as those of the Department of Motor Vehicles (Kendler & Prescott, 2006). The first cohort was a population-based sample of same-sex female–female (FF) twin pairs born between 1934 and 1974. The second cohort consisted of male–male/male–female twins (MF) of pairs born between 1940 and 1974. FF twins were assessed at four different times whereas the MF sample was interviewed twice. In this study, we used data from the face-to-face from the first assessment interview for the FF sample (FF1; *n* = 2162, M age = 30.1 years, s.d. = 7.6) conducted between January 1987 and July 1989 and the first MF assessment conducted between March 1993 and October 1996 (MF1; *n* = 6843, 75% male; M age = 35.5 years, s.d. = 9.2, female M age = 35.4 years, s.d. = 9.0) for a total sample size of *N* = 9005 twins.

Zygosity, which was assessed by a combination of standard questionnaire items and photographs validated against DNA-based measures, with an estimated error rate of under 2%, produced the following five twin groups: complete same-sex MZ pairs (*n* = 1461), incomplete same-sex MZ (*n* = 285), complete same-sex DZ (*n* = 1079), incomplete same-sex DZ (*n* = 338), complete opposite-sex pairs (*n* = 1411), and incomplete opposite-sex (*n* = 476). Four individual twins were missing a zygosity classification. Ethical approval was granted from the Institutional Review Board at Virginia Commonwealth University. Both the complete pairs and the unpaired twins were included in our analyses.

### Assessment of depressive criteria

Using a structured interview based on the Structured Clinical Interview for DSM-III-R (Spitzer & Williams, 1985) modified to include DSM-IV criteria, the presences of each of the 14 disaggregated diagnostic symptom criteria for major depressive disorder (MDD) were assessed for the year prior to the interview. Interviewers inquired whether each symptom was related to an illness or taking of any medications and how much the symptom interfered with their daily activities. Only positively reported symptoms that were not considered to be related to illness or medication use were retained.

All items were codes as binary (0 = absent, 1 = present). The disaggregated MDD symptom criteria are: (1) depressed mood, (2) anhedonia, (3) weight decrease, (4) weight increase, (5) appetite decrease, (6) appetite increase, (7) insomnia, (8) hypersomnia, (9) psychomotor agitation, (10) psychomotor retardation, (11) fatigue, (12) difficulty concentrating, (13) feelings of guilt/worthlessness, and (14) thoughts of self-harm/suicidal ideation. For this study, the disaggregated weight/appetite, sleep, and psychomotor problem symptoms were collapsed by creating a single binary-coded positive (1) if any of separate disaggregated items were positively endorsed to obtain the nine official DSM MDD criteria.

In a follow-up section, twins were then asked how many of these positively endorsed symptoms had occurred temporally to ensure that the set formed a syndrome and were not experienced haphazardly across the year. For each twin, it was possible to form two possible separate syndromes based on the timing of cooccurrences of symptom sets.

### Symptom classification

For the analyses in this study, a classification algorithm was developed to assign individual MD symptoms to either an ‘IN’ or ‘OUT’ grouping. A symptom was assigned to the ‘IN’ group if it was experienced with other symptoms that were part of a syndromal episode that algorithmically met full criteria for an MD DSM-IV diagnosis. A symptom was assigned to the ‘OUT’ group if the symptom was experienced over the year prior to the interview but was not part of a syndromal symptom clustering that satisfied the threshold for an MD diagnosis. Since it was possible for a twin to report symptom information for more than a single syndrome (a second was assessed in the interview), it was possible that an individual MD symptom could be classified in both the ‘IN’ and ‘OUT’ groupings for the same twin. For example, if for one of the reported episodes, the aggregated sleep symptom was endorsed with a complement of other symptoms that met DSM threshold for MD, it would be assigned to the ‘IN’ group. If, however, for a second separate episode the sleep symptom was endorsed but did not co-occur with enough other DSM depressive criteria, this same symptom would also be assigned to the ‘OUT’ group for the same individual.

### Statistical analysis

Univariate and bivariate ACE variance component estimation models (where A stands for additive genetic effects, C for common or shared environment and E for individual-specific environment) were fitted to the nine ‘IN’ and ‘OUT’ classified MD symptom items. ACE models were specified as variance components rather than Cholesky path decompositions. The benefit of the variance component estimation approach is that it mitigates possible biasing of parameter estimates due to the implicit bounding in the Cholesky decomposition that can result in departures of type I errors from their theoretical expectations under the null hypothesis(Verhulst, Prom-Wormley, Keller, Medland, & Neale, 2019). ACE models decompose the observed phenotypic correlation within and cross-trait correlations between MZ and DZ twins into additive genetic, shared, and unique environmental sources of variance. Thus, these models infer the magnitude of these sources of variance – including additive genetic effects – from patterns of twin correlations. All models were fit within the R computing environment 4.2.2 (Team, 2022) using the R packages ‘OpenMx’ (Neale et al., 2016) version 2.20.7 and ‘umx’ version 4.15.1 (Bates, Neale, & Maes, 2019). The best-fitting model, which reflected the optimal balance of model complexity and explanatory power, was chosen by Akaike's Information Criterion (AIC) (Akaike, 1987). Statistical significance is assessed by the conservative approach of non-overlapping 95% confidence intervals (CIs).

## Results

### Descriptive results

Table 1 presents key descriptive summaries for each of the ‘In’ and ‘Out’ symptom classification in our sample. All nine DSM MD criteria were reported more frequently outside than inside of depressive episodes. Outside of episodes, the four most common reported criteria were depressed mood, weight loss or gain, fatigue, and sleep problems. Within depressive episodes, the order was somewhat different: depressed mood, loss of interest, psychomotor problems, and sleep problems. The rarest symptom both in and out of depressive episodes was thoughts of death.

**Table 1.** Descriptive results of depressive criteria occurring ‘In’ *v.* ‘Out’ of major depressive episodes

DSM-IV criterion	Brief description	Short term	‘In’ *v.* ‘Out’	Count	Proportion	Std error
A.1	Depressed mood	DM	In	995	0.111	0.003
			Out	2508	0.279	0.005
A.2	Loss of interest or pleasure	LIP	In	906	0.101	0.003
			Out	1609	0.179	0.004
A.3	Weight loss or gain	WP	In	782	0.087	0.003
			Out	2364	0.263	0.005
A.4	Insomnia or hypersomnia	SP	In	824	0.092	0.003
			Out	1694	0.189	0.004
A.5	Psychomotor agitation or retardation	PP	In	814	0.091	0.003
			Out	1855	0.207	0.004
A.6	Fatigue	FA	In	778	0.087	0.003
			Out	1863	0.208	0.004
A.7	Feeling worthless	FW	In	612	0.068	0.003
			Out	964	0.107	0.003
A.8	Difficulty concentrating	DC	In	611	0.068	0.003
			Out	921	0.103	0.003
A.9	Thoughts of death	TD	In	275	0.031	0.002
			Out	861	0.096	0.003

Sample *N*: 8978–8981 due to variation in missing data; WP, weight problems; SP, sleep problems; PP, psychomotor problems.

**Figure 1.**
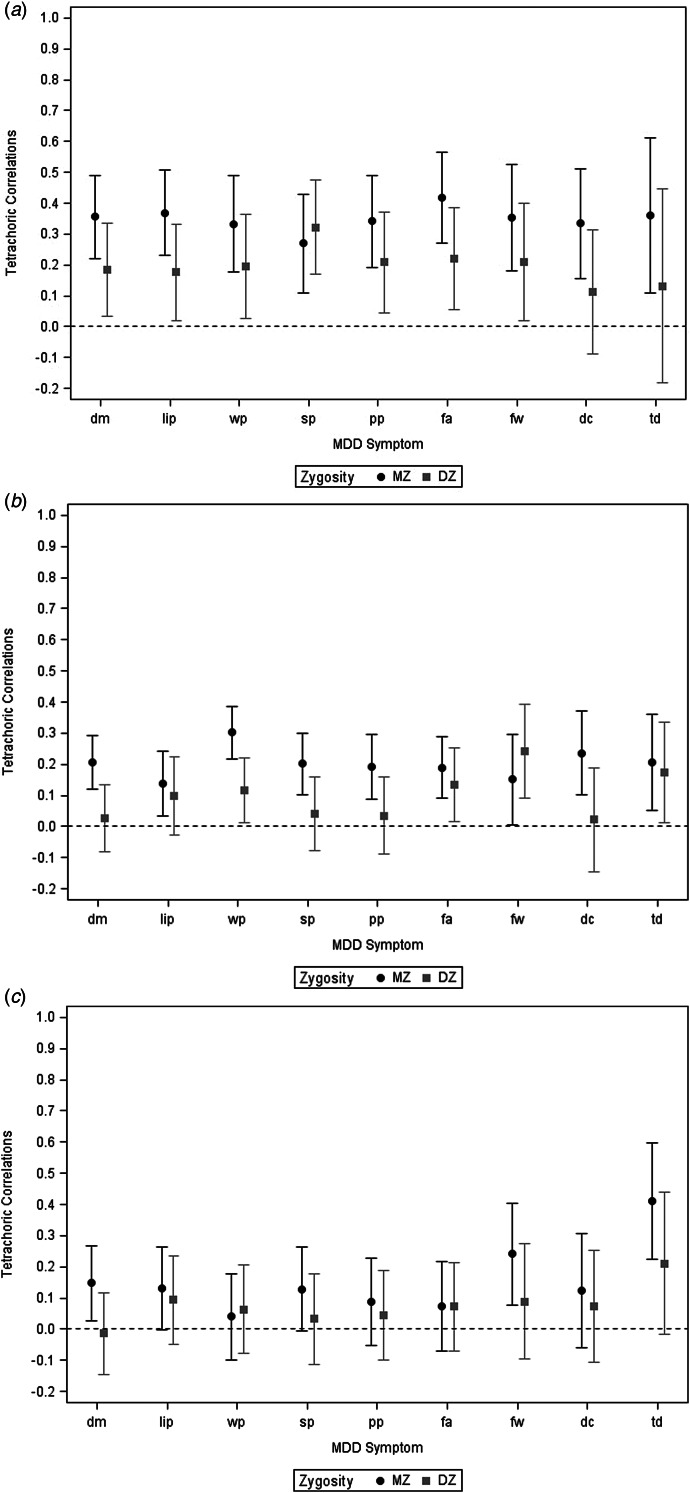
(*a*) Tetrachoric correlations in monozygotic (MZ) (black circles) and dizygotic (DZ) (grey squares) twins for DSM major depression criteria occurring within major depressive episodes. The *y*-axis represents the value of the correlation. The *x*-axis represents the nine DSM MD symptomatic criteria. For abbreviations, see Table 1. (*b*) Tetrachoric correlations in monozygotic (MZ) (black circles) and dizygotic (DZ) (grey squares) twins for DSM major depression criteria occurring outside of major depressive episodes. The *y*-axis represents the value of the correlation. The *x*-axis represents the nine DSM MD symptomatic criteria. For abbreviations, see Table 1. (*c*) Tetrachoric correlations in monozygotic (MZ) (black circles) and dizygotic (DZ) (grey squares) twins for DSM major depression criteria occurring within major depressive episodes in one twin and outside of major depressive episodes in the cotwin. The *y*-axis represents the value of the correlation. The *x*-axis represents the nine DSM MD symptomatic criteria. For abbreviations, see Table 1.

Figures 1*a*–*c* present tetrachoric correlations and 95% CIs in MZ and DZ twins for, respectively, the in-MD symptoms, the out-MD symptoms, and the cross-correlations for in-MD symptoms in one twin and the out-MD symptom in the cotwin. For the exact correlations and CIs see online Supplementary Appendix Tables 1a–1c. The mean (±95% CIs) tetrachoric correlations across the nine criteria for the in–in, out–out, and in–out analyses are seen in the top section of Table 2. By a paired *t* test on the Fisher *z*-transformed correlations, the mean correlations for the nine DSM MD criteria in the in–in MZ pairs (0.35) were substantially and significantly higher than observed in the MZ pairs for the out–out criteria (0.20) (*t* = 0.15, *p* < 0.01). A similar pattern, also significant was seen in the DZ pairs: 0.20 *v.* 0.07 (*t* = 0.10, *p* < 0.02).

**Table 2.** Key parameter estimates

Description	Sample/variable	Estimate	Lower 95% CI	Upper 95% CI
In–In criteria mean tetrachoric	MZ twins	0.35	0.32	0.38
	DZ twins	0.20	0.15	0.24
Out–Out criteria mean tetrachoric	MZ twins	0.20	0.17	0.24
	DZ twins	0.07	0.04	0.16
In–Out criteria mean tetrachoric	MZ twins	0.15	0.07	0.24
	DZ twins	0.07	0.03	0.12
Mean modeling estimates for In criteria	a^2^	0.31	0.22	0.41
	c^2^	0.05	−0.01	0.12
	e^2^	0.64	0.61	0.66
Mean modeling estimates for Out criteria	a^2^	0.15	0.08	0.21
	c^2^	0.05	−0.01	0.10
	e^2^	0.80	0.77	0.84
Mean genetic correlation for In and Out criteria	r_g_	0.07	−0.07	0.21

A, additive genetic effect; c, shared family environment; e, individual specific environment; r_g,_ genetic correlation; CI, confidence interval.

With the exception of feelings of worthless and thoughts of death, the cross-correlations between the IN criteria in one twin with the OUT criteria in their cotwin were low (Fig. 1*c*) with the mean correlations equal to 0.15 and 0.07 in MZ and DZ pairs, respectively (bottom section Table 2).

### Model fitting

**Figure 2.**
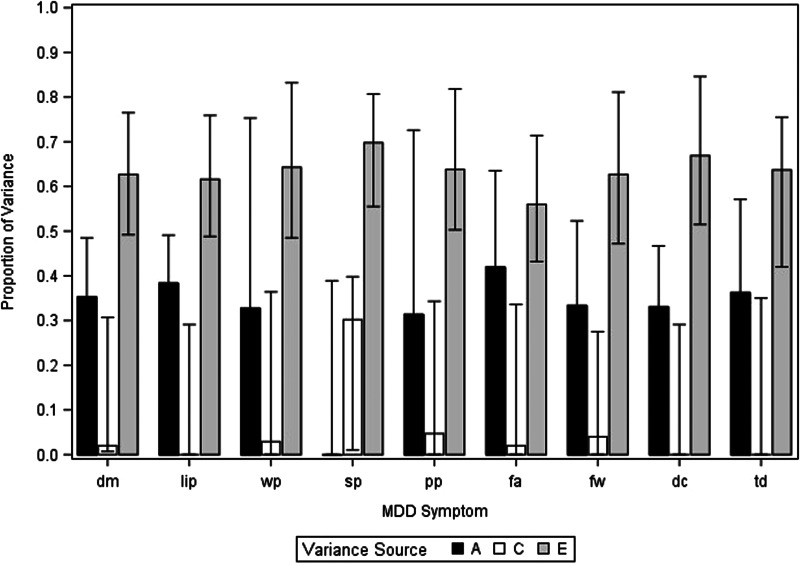
Estimates of additive genetic effects (a^2^), shared environmental effects (c^2^), and individual-specific environmental effects (e^2^) for nine DSM symptomatic criteria for MD occurring within major depressive episodes. These results are from a full ACE univariate twin model. For abbreviations, see Table 1.

We present, in online Supplementary Appendix Table 2, the full model-fitting results for the in–in depressive criteria. For these analyses, AE models fitted best for all criteria except sleep problems. However, given concerns about parameter biases in such reduced models (Sullivan & Eaves, 2002), we present in Fig. 2*a* the results of the full ACE model. As can be seen, with the exception of sleep problems, the estimates of the heritability of all individual depressive criteria varied from 0.31 for psychomotor changes to 0.42 for fatigue. The mean estimates for a^2^, c^2^, and e^2^ for the nine In depressive criteria are seen in Table 2. The mean heritability was estimated at +0.31 (0.22–0.41).

Online Supplementary Appendix Table 3 presents model-fitting results for the out–out depressive criteria. AE models fitted best for all criteria except feelings of worthlessness and suicidal ideation. We present results of a full ACE model in Fig. 2*b*. With the exception of feelings of worthlessness, heritability estimates were modest, ranging from 0.09 for loss of interest to 0.29 for weight changes. The mean estimates for a^2^, c^2^, and e^2^ for the nine Out depressive criteria are also seen in Table 2, with heritability estimated at +0.15 (0.08–0.21). Comparing the key estimates for the in–in *v.* out–out twin models averaged across all nine criteria, the heritability is significantly higher in the in–in analyses while the proportion of variance in liability due to individual-specific environmental factors (e^2^) was significantly higher in the out–out analyses.

**Figure 3.**
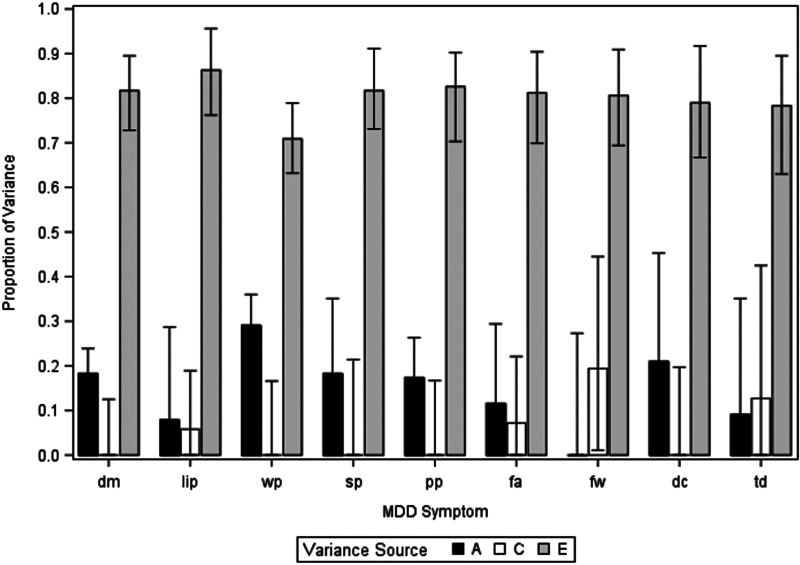
Estimates of additive genetic effects (a^2^), shared environmental effects (c^2^), and individual-specific environmental effects (e^2^) for nine DSM symptomatic criteria for MD occurring outside of major depressive episodes. These results are from a full ACE univariate twin model. For abbreviations, see Table 1.

Finally, as seen in detail in online Supplementary Appendix Table 4, we fitted, for the sake of simplicity, only ACE, AE, and CE models for the bivariate In–Out analyses. The AE model fitted best for all nine criteria. We present in Fig. 3 the estimated genetic correlations between the in and out DSM criteria. These ranged from −0.18 for weight changes and concentration problems to +0.31 for psychomotor changes. Only one of these genetic correlations – for psychomotor changes – was statistically significant. The mean genetic correlation (±95% CIs) across the nine criteria was +0.07 and was not statistically different from zero (Table 1).

## Discussion

We sought in these analyses to understand, from a genetic–epidemiologic perspective, the relationship of individual symptomatic criteria for MD when they are reported as occurring in the midst of a depressive episode *v.* outside such an episode. This question is of interest because it directly addresses the relationship between the two views of *depression* articulated above, that is as (i) a syndromal DSM diagnosis and (ii) current depressive symptoms. We were particularly interested in distinguishing between two hypotheses about the association between these two ‘kinds’ of depression – the convergent and divergent perspectives – with respect to psychiatric genetics research.

Of the numerous results presented here, three are of particular importance. First, we found, in a population-based sample of twins, interviewed by trained mental health professionals, that all nine depressive criteria, assessed in the last year, were considerably more common outside of than within depressive episodes. This was even true for thoughts of death/suicide, typically considered the most severe of the depressive criteria. This finding, in and of itself, should give pause to advocates for a close relationship between depressive symptoms and syndromal MD in that they suggest that there are many other causes for the occurrence of individual MD criteria in the general population that are not related to an MDD.

Second, the familial influences on depressive criteria were considerably stronger when those criteria occurred within *v.* outside of a depressive episode. We saw this in the correlations of the depressive criteria in both MZ and DZ pairs. Then we tested the magnitude of genetic effects of these criteria by simple twin modeling. Estimates of heritability were overall substantially higher for depressive criteria reported within rather than outside a depressive episode. Another result of our modeling, perhaps of even greater interest, is that the role of individual-specific environmental factors – for example, possible stressors occurring to one twin but not the other – contributed substantially more to the depressive criteria reported outside compared to those within depressive episodes. This would be consistent with the concern that a range of environmental events – back pain, a crying baby or a noisy next-door party causing insomnia, a tough work schedule or ill relatives leading to tiredness or difficulty concentrating – are more likely to influence depressive symptoms that are experienced on their own rather than as part of a full depressive syndrome.

Third, the single best test of the convergent and divergent perspectives on the two versions of depression is the genetic correlation between our criteria that occur within and outside of MD episodes. Although our sample is not well powered to obtain precise estimates of genetic correlations, our results were nonetheless clear. Genetic correlations for the nine DSM-IV criteria for MD when they occurred within *v.* outside depressive episodes were uniformly quite low.

In aggregate, our findings provide consistent support for the divergent viewpoint about the relationship between self-report current depressive symptoms and last-year or lifetime MD. They suggest that using, in a genetic design, current depressive symptoms as a proxy for lifetime MD, is likely to be problematic as the two constructs have only quite modestly correlated genetic risks. Furthermore, the relatively large estimates for the variance of the out depressive criteria explained by e^2^ – individual specific environment – is consistent with the concern that relatively random personal factors (e.g. tiredness due to overwork or extended childcare, sleep difficulties due to ill children, back pain or a snoring spouse) play a major role in risk for depressive symptoms in general population samples occurring outside of depressive episodes.

Our results appear to be inconsistent with multiple reports of high genetic correlations, based on genome-wide association study-derived polygenic risk scores, between lifetime MD and various forms of current depressive symptoms with representative estimates from three studies of 0.70 (Turley et al., 2018), 1.00 (Direk et al., 2017) and 0.72–0.88 across different cohorts (Levey et al., 2021). We have no fully adequate explanation for this discrepancy, although the methods of determining the genetic relationship between these two constructs of *depression* are quite methodologically distinct. Our method addressed a more focused question, in a single study sample: the temporal relationship between DSM criteria. This approach allowed us to control for many background features such as sample composition, nature of assessments (e.g. interview *v.* questionnaire) and instrument differences that commonly occur in the calculation of a genetic correlations. Furthermore, genetic correlations are statistically complex with a range of assumptions and the possibility of sources of upward bias (van Rheenen, Peyrot, Schork, Lee, & Wray, 2019) including, for example, assortative mating (Border et al., 2022) and the use of super-normal controls (Kendler, Chatzinakos, & Bacanu, 2020).

### Limitations

The results presented here should be interpreted in the context of five potential methodological limitations. First, our sample is restricted to white, native-born Virginia twins and might not extrapolate to other samples. Second, the reliability of interview-based assessments of MD has been questioned (Regier et al., 2013). However, we performed a test–retest study in VATSPUD for lifetime MD in 375 individuals with assessment an average of 30 days apart and obtained a solid *κ* statistic (Cohen, 1960) of +0.66 (Kendler & Prescott, 2006). Third, individual criteria for MD assessed in the last year in VATSPSUD were only recorded if they were not the result of illness or prescribed medications. This is not a requirement in any of the self-report depressive symptom questionnaires with which we are familiar. However, it seems likely that this screening would, in anything, reduce the differences between in and out depressive symptoms as episodes of tiredness due to the flu or difficulty sleeping from an acute exacerbation of arthritis would have been ruled out.

Fourth, given problems with self-reporting, it could be argued that a depressive criterion that co-occurred with three other criteria (thus having four rather than the required five symptomatic criteria) might be considered to indicate a full MD syndrome. Therefore, we re-ran our main analyses censoring out as ‘unknown’ individual criteria that co-occurred with three others. We present in the online Supplementary Appendix Fig. 1 the resulting twin correlations between the in and out criterion. A slight decline was observed in the level for most of the out symptoms compared to those reported here.

Fifth, it could be argued that our study would be more relevant to current genetics research if we had assessed lifetime MD, as this is the measure most often used in genetic studies. However, in the design of our interview, we focused on the details of depressive symptomatology over the last year because we felt the memory burdens of recalling and dating accurately all episodes of common symptoms such as tiredness, insomnia, and difficulties concentrating over a lifetime were unrealistic.

## Conclusions

We sought to examine, using a novel classification scheme, the relationship between DSM depressive criteria that occur temporally within *v.* outside depressive episodes. This question is of interest because it addresses the fraught question of the etiological relationship between two divergent constructs of *depression* common in the mental health literature: current depressive symptoms and lifetime (or last year) MD. We suggested two alternative views of these two constructs: convergent (where the differences are only quantitative) and divergent (where the differences are qualitative). Our results support the divergent hypothesis. Depressive criteria reported in the last year outside of depressive episodes were considerably less familial, had a lower heritability, and had at most a modest genetic correlation with the same criteria occurring with depressive episodes. Our results, which should be treated at tentative pending replication, suggest considerable caution is indicated when considering using current depressive symptoms as a proxy measure for lifetime or last year MD.

## Supporting information

Kendler and Aggen supplementary material Kendler and Aggen supplementary material

## Supplementary material

The supplementary material for this article can be found at https://doi.org/10.1017/S0033291723001241.
