# Supplementary material for: A population-based twin study of the symptomatic diagnostic criteria for major depression that occur within versus outside of major depressive episodes
Source: Psychol Med. 2023 May 8;53(15):7458–65. doi: 10.1017/S0033291723001241 (PMC10719671; doi:10.1017/S0033291723001241)

Appendix

Table 1a

Tetrachoric correlations and 95% confidence intervals (CIs) in MZ and DZ twins for the In-MD Criteria

| Criteria | Zygosity | Correlation | Lower CL | Upper CL |
| --- | --- | --- | --- | --- |
| dm | MZ | 0.357 | 0.222 | 0.492 |
| dm | DZ | 0.185 | 0.035 | 0.335 |
| lip | MZ | 0.370 | 0.230 | 0.509 |
| lip | DZ | 0.176 | 0.020 | 0.333 |
| wp | MZ | 0.333 | 0.176 | 0.490 |
| wp | DZ | 0.195 | 0.027 | 0.364 |
| sp | MZ | 0.270 | 0.110 | 0.430 |
| sp | DZ | 0.322 | 0.169 | 0.475 |
| pp | MZ | 0.342 | 0.194 | 0.491 |
| pp | DZ | 0.208 | 0.046 | 0.371 |
| fa | MZ | 0.417 | 0.270 | 0.564 |
| fa | DZ | 0.222 | 0.056 | 0.387 |
| fw | MZ | 0.353 | 0.181 | 0.526 |
| fw | DZ | 0.210 | 0.019 | 0.401 |
| dc | MZ | 0.334 | 0.157 | 0.512 |
| dc | DZ | 0.113 | -0.086 | 0.312 |
| td | MZ | 0.360 | 0.110 | 0.611 |
| td | DZ | 0.132 | -0.182 | 0.447 |

Table 1b

Tetrachoric correlations and 95% confidence intervals (CIs) in MZ and DZ twins for the Out-MD Criteria

| Criteria | Zygosity | Correlation | Lower CL | Upper CL |
| --- | --- | --- | --- | --- |
| dm | MZ | 0.207 | 0.120 | 0.294 |
| dm | DZ | 0.026 | -0.081 | 0.133 |
| lip | MZ | 0.138 | 0.034 | 0.242 |
| lip | DZ | 0.098 | -0.028 | 0.223 |
| wp | MZ | 0.302 | 0.218 | 0.387 |
| wp | DZ | 0.117 | 0.011 | 0.222 |
| sp | MZ | 0.202 | 0.103 | 0.301 |
| sp | DZ | 0.040 | -0.079 | 0.159 |
| pp | MZ | 0.192 | 0.089 | 0.295 |
| pp | DZ | 0.036 | -0.088 | 0.159 |
| fa | MZ | 0.189 | 0.091 | 0.287 |
| fa | DZ | 0.134 | 0.016 | 0.252 |
| fw | MZ | 0.151 | 0.007 | 0.295 |
| fw | DZ | 0.242 | 0.093 | 0.391 |
| dc | MZ | 0.237 | 0.103 | 0.370 |
| dc | DZ | 0.023 | -0.144 | 0.190 |
| td | MZ | 0.207 | 0.051 | 0.363 |
| td | DZ | 0.175 | 0.013 | 0.337 |

Table 1c

Tetrachoric correlations and 95% confidence intervals (CIs) in MZ and DZ twins for the cross correlations for in-MD symptoms in one twin and the out-MD symptom in the cotwin

| Criteria | Zygosity | Correlation | Lower CL | Upper CL |
| --- | --- | --- | --- | --- |
| dm | MZ | 0.148 | 0.029 | 0.268 |
| dm | DZ | -0.014 | -0.146 | 0.118 |
| lip | MZ | 0.131 | -0.003 | 0.265 |
| lip | DZ | 0.094 | -0.049 | 0.236 |
| wp | MZ | 0.040 | -0.097 | 0.177 |
| wp | DZ | 0.063 | -0.079 | 0.205 |
| sp | MZ | 0.128 | -0.007 | 0.262 |
| sp | DZ | 0.033 | -0.113 | 0.178 |
| pp | MZ | 0.089 | -0.050 | 0.228 |
| pp | DZ | 0.045 | -0.099 | 0.189 |
| fa | MZ | 0.072 | -0.071 | 0.215 |
| fa | DZ | 0.072 | -0.071 | 0.215 |
| fw | MZ | 0.241 | 0.077 | 0.405 |
| fw | DZ | 0.089 | -0.096 | 0.274 |
| dc | MZ | 0.123 | -0.061 | 0.306 |
| dc | DZ | 0.073 | -0.106 | 0.252 |
| td | MZ | 0.411 | 0.226 | 0.597 |
| td | DZ | 0.211 | -0.017 | 0.439 |

Table 2: Model fitting results for univariate ACE model for 9 “IN” classified MDD symptoms

| base | comparison | ep | minus2LL | df | AIC | Diff LL | Diff df | p |
| --- | --- | --- | --- | --- | --- | --- | --- | --- |
| iDM | -- | 4 | 3552 | 5682 | -7812 | . | . | . |
| iDM | idmAE | 3 | 3552 | 5683 | -7814 | -0.000 | 1 | 1.000 |
| iDM | idmCE | 3 | 3555 | 5683 | -7811 | 3.622 | 1 | 0.057 |
| iLIP | -- | 4 | 3333 | 5682 | -8031 | . | . | . |
| iLIP | ilipAE | 3 | 3333 | 5683 | -8033 | 0.000 | 1 | 1.000 |
| iLIP | ilipCE | 3 | 3337 | 5683 | -8029 | 4.042 | 1 | 0.044 |
| iwp | -- | 4 | 2980 | 5682 | -8384 | . | . | . |
| iwp | iwpAE | 3 | 2980 | 5683 | -8386 | 0.026 | 1 | 0.873 |
| iwp | iwpCE | 3 | 2982 | 5683 | -8384 | 1.956 | 1 | 0.162 |
| isp | -- | 4 | 3065 | 5681 | -8297 | . | . | . |
| isp | ispAE | 3 | 3069 | 5682 | -8295 | 3.745 | 1 | 0.053 |
| isp | ispCE | 3 | 3065 | 5682 | -8299 | 0.000 | 1 | 1.000 |
| ipp | -- | 4 | 3126 | 5682 | -8238 | . | . | . |
| ipp | ippAE | 3 | 3126 | 5683 | -8240 | 0.071 | 1 | 0.790 |
| ipp | ippCE | 3 | 3128 | 5683 | -8238 | 1.983 | 1 | 0.159 |
| ifa | -- | 4 | 2932 | 5682 | -8432 | . | . | . |
| ifa | ifaAE | 3 | 2932 | 5683 | -8434 | 0.000 | 1 | 1.000 |
| ifa | ifaCE | 3 | 2936 | 5683 | -8430 | 4.111 | 1 | 0.043 |
| ifw | -- | 4 | 2494 | 5682 | -8870 | . | . | . |
| ifw | ifwAE | 3 | 2494 | 5683 | -8872 | 0.037 | 1 | 0.848 |
| ifw | ifwCE | 3 | 2496 | 5683 | -8870 | 1.637 | 1 | 0.201 |
| idc | -- | 4 | 2533 | 5682 | -8831 | . | . | . |
| idc | idcAE | 3 | 2533 | 5683 | -8833 | 0.000 | 1 | 1.000 |
| idc | idcCE | 3 | 2536 | 5683 | -8830 | 2.833 | 1 | 0.092 |
| itd | -- | 4 | 1472 | 5682 | -9892 | . | . | . |
| itd | itdAE | 3 | 1472 | 5683 | -9894 | -0.000 | 1 | 1.000 |
| itd | itdCE | 3 | 1473 | 5683 | -9893 | 1.413 | 1 | 0.235 |
| “I” stands for “In.” LL – loglikelihood. Diff – difference in; DF – degrees of freedom. For other abbreviations, see the manuscript table 1. | | | | | | | | |

Table 2: Model fitting results for univariate ACE model for 9 “OUT” classified MDD symptoms

| base | comparison | ep | minus2LL | df | AIC | Diff LL | Diff df | p |
| --- | --- | --- | --- | --- | --- | --- | --- | --- |
| oDM | -- | 4 | 6708 | 5682 | -4656 | . | . | . |
| oDM | odmAE | 3 | 6708 | 5683 | -4658 | 0.000 | 1 | 1.000 |
| oDM | odmCE | 3 | 6713 | 5683 | -4653 | 4.725 | 1 | 0.030 |
| olip | -- | 4 | 5383 | 5682 | -5981 | . | . | . |
| olip | olipAE | 3 | 5383 | 5683 | -5983 | 0.174 | 1 | 0.677 |
| olip | olipCE | 3 | 5383 | 5683 | -5983 | 0.226 | 1 | 0.634 |
| owp | -- | 4 | 6577 | 5682 | -4787 | . | . | . |
| owp | owpAE | 3 | 6577 | 5683 | -4789 | 0.000 | 1 | 0.999 |
| owp | owpCE | 3 | 6584 | 5683 | -4782 | 6.874 | 1 | 0.009 |
| osp | -- | 4 | 5726 | 5681 | -5636 | . | . | . |
| osp | ospAE | 3 | 5726 | 5682 | -5638 | 0.000 | 1 | 1.000 |
| osp | ospCE | 3 | 5730 | 5682 | -5634 | 3.371 | 1 | 0.066 |
| opp | -- | 4 | 5486 | 5682 | -5878 | . | . | . |
| opp | oppAE | 3 | 5486 | 5683 | -5880 | 0.000 | 1 | 1.000 |
| opp | oppCE | 3 | 5489 | 5683 | -5877 | 2.892 | 1 | 0.089 |
| ofa | -- | 4 | 5696 | 5682 | -5668 | . | . | . |
| ofa | ofaAE | 3 | 5696 | 5683 | -5670 | 0.302 | 1 | 0.583 |
| ofa | ofaCE | 3 | 5697 | 5683 | -5669 | 0.552 | 1 | 0.458 |
| ofw | -- | 4 | 3654 | 5682 | -7710 | . | . | . |
| ofw | ofwAE | 3 | 3657 | 5683 | -7709 | 2.957 | 1 | 0.086 |
| ofw | ofwCE | 3 | 3654 | 5683 | -7712 | -0.000 | 1 | 1.000 |
| odc | -- | 4 | 3749 | 5682 | -7615 | . | . | . |
| odc | odcAE | 3 | 3749 | 5683 | -7617 | 0.000 | 1 | 1.000 |
| odc | odcCE | 3 | 3752 | 5683 | -7614 | 2.778 | 1 | 0.096 |
| otd | -- | 4 | 3294 | 5682 | -8070 | . | . | . |
| otd | otdAE | 3 | 3295 | 5683 | -8071 | 0.501 | 1 | 0.479 |
| otd | otdCE | 3 | 3294 | 5683 | -8072 | 0.157 | 1 | 0.691 |
| “O” stands for “out.” LL – loglikelihood. Diff – difference in; DF – degrees of freedom. For other abbreviations, see the manuscript table 1. | | | | | | | | |

Table 3: Model fitting results for bivariate ACE model for the 9 “IN-OUT” paired MDD symptoms

| base | comparison | ep | minus2LL | df | AIC | Diff LL | Diff df | p |
| --- | --- | --- | --- | --- | --- | --- | --- | --- |
| ioDM | -- | 11 | 9906 | 11369 | 9928 | . | . | . |
| ioDM | iodmAE | 8 | 9908 | 11372 | 9924 | 2.070 | 3 | 0.558 |
| ioDM | iodmCE | 8 | 9917 | 11372 | 9933 | 10.990 | 3 | 0.012 |
| iolip | -- | 11 | 8546 | 11369 | 8568 | . | . | . |
| iolip | iolipAE | 8 | 8547 | 11372 | 8563 | 0.531 | 3 | 0.912 |
| iolip | iolipCE | 8 | 8555 | 11372 | 8571 | 8.306 | 3 | 0.040 |
| iowp | -- | 11 | 9462 | 11369 | 9484 | . | . | . |
| iowp | iowpAE | 8 | 9466 | 11372 | 9482 | 4.040 | 3 | 0.257 |
| iowp | iowpCE | 8 | 9471 | 11372 | 9487 | 9.020 | 3 | 0.029 |
| iosp | -- | 11 | 8675 | 11367 | 8697 | . | . | . |
| iosp | iospAE | 8 | 8679 | 11370 | 8695 | 4.650 | 3 | 0.200 |
| iosp | iospCE | 8 | 8681 | 11370 | 8697 | 6.660 | 3 | 0.084 |
| iopp | -- | 11 | 8529 | 11369 | 8551 | . | . | . |
| iopp | ioppAE | 8 | 8533 | 11372 | 8549 | 4.090 | 3 | 0.252 |
| iopp | ioppCE | 8 | 8544 | 11372 | 8560 | 14.730 | 3 | 0.002 |
| iofa | -- | 11 | 8514 | 11369 | 8536 | . | . | . |
| iofa | iofaAE | 8 | 8517 | 11372 | 8533 | 2.370 | 3 | 0.499 |
| iofa | iofaCE | 8 | 8520 | 11372 | 8536 | 5.340 | 3 | 0.148 |
| iofw | -- | 11 | 6097 | 11369 | 6119 | . | . | . |
| iofw | iofwAE | 8 | 6100 | 11372 | 6116 | 3.560 | 3 | 0.313 |
| iofw | iofwCE | 8 | 6102 | 11372 | 6118 | 5.240 | 3 | 0.155 |
| iodc | -- | 11 | 6266 | 11369 | 6288 | . | . | . |
| iodc | iodcAE | 8 | 6269 | 11372 | 6285 | 2.760 | 3 | 0.430 |
| iodc | iodcCE | 8 | 6273 | 11372 | 6289 | 7.310 | 3 | 0.063 |
| iotd | -- | 11 | 4715 | 11369 | 4737 | . | . | . |
| iotd | iotdAE | 8 | 4717 | 11372 | 4733 | 1.820 | 3 | 0.611 |
| iotd | iotdCE | 8 | 4717 | 11372 | 4733 | 1.470 | 3 | 0.690 |
| “I” stands for “in”; “O” stands for “out.” LL – loglikelihood. Diff – difference in; DF – degrees of freedom. For other abbreviations, see the manuscript table 1. | | | | | | | | |

Figure 1: MZ and DZ twin tetrachoric correlations with 95% CIs for 9 OUT-OUT MD criteria (dropping positive OUT criteria that co-occurred in a temporal cluster with 3 other MD criteria)


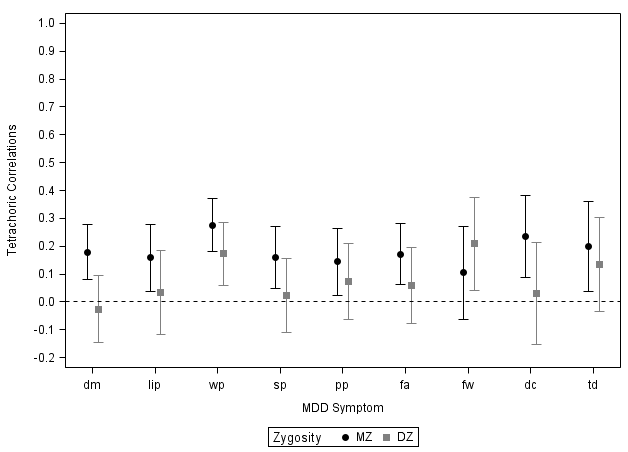

Supplement: Kendler and Aggen supplementary material [file S0033291723001241sup001.docx]
